# Supplementary material for: Injury-related cell death and proteoglycan loss in articular cartilage: Numerical model combining necrosis, reactive oxygen species, and inflammatory cytokines
Source: PLoS Comput Biol. 2023 Jan 26;19(1):e1010337. doi: 10.1371/journal.pcbi.1010337 (PMC9879441; doi:10.1371/journal.pcbi.1010337)
Supplement: S1 Text — More detailed explanation of the biomechanical material model. (DOCX) [file pcbi.1010337.s001.docx]

**Electronic Supplementary material**

For “*Injury-related cell death and proteoglycan loss in articular cartilage: Numerical model combining necrosis, reactive oxygen species, and inflammatory cytokines*” (in *PLOS Computational Biology*) by

Joonas P. Kosonen^1^, Atte S.A. Eskelinen^1^, Gustavo A. Orozco^1,2^, Petteri Nieminen^3^, Donald D. Anderson^4^, Alan J. Grodzinsky^5^, Rami K. Korhonen^1^, and Petri Tanska^1^

^1^*Department of Technical Physics, University of Eastern Finland, Kuopio, Finland*

*^2^Department of Biomedical Engineering, Lund University, Lund, Sweden*

*^3^Institute of Biomedicine, University of Eastern Finland, Kuopio, Finland,*

*^4^Departments of Orthopedics & Rehabilitation and Biomedical Engineering, University of Iowa, Iowa City, IA, USA*

^5^*Departments of Biological Engineering, Electrical Engineering and Computer Science and Mechanical Engineering, Massachusetts Institute of Technology, Cambridge, MA, USA*

**Corresponding author:**

*Joonas P. Kosonen

Department of Applied Physics

University of Eastern Finland

Yliopistonranta 1 F

POB 1627, Kuopio FI-70211, Finland

Tel: +358 50 3043474

[joonas.kosonen@uef.fi](mailto:attees@uef.fi)

**S1 Text. Biomechanical material model**

Cartilage was modeled as a fibril-reinforced porohyperelastic material model with Donnan osmotic swelling and chemical expansion (FRPHES). Previously, the FRPHES material model has been successfully used to simulate the experimentally observed cartilage behavior after biomechanical compression [1–4]. In this material model, the cartilage is considered as an anisotropic biphasic material including a solid phase with fibrillar (collagen) and non-fibrillar (proteoglycans, PG) components and a fluid phase. Furthermore, the material model considers depth-dependent PG (fixed charge density, FCD) and water content as well as depth-wise collagen distribution and orientation (see S1 Table) [1,3].

Cauchy stress tensor of a Neo-Hookean solid material was used to describe stresses in the non-fibrillar solid matrix $\boldsymbol{\sigma}_{\mathrm{nfb}}$ [3,5]:

|  | $\boldsymbol{\sigma}_{\mathrm{nfb}}=K_{\mathrm{nfb}}\frac{\ln\left( J \right)}{J}\mathbf{I}+\frac{G_{\mathrm{nfb}}}{J}\left( \mathbf{F}\mathbf{F}^{T}-J^{\frac{2}{3}} \mathbf{I} \right),$ | (S1) |
| --- | --- | --- |

where $\mathbf{F}$ is the deformation gradient tensor, $\mathbf{I}$ is the unit tensor and $J=\det\left( \mathbf{F} \right)$ is the volumetric deformation, i.e., determinant of the deformation gradient tensor. The bulk $K_{\mathrm{nfb}}$ and shear moduli $G_{\mathrm{nfb}}$ of the non-fibrillar matrix were determined as

|  | $K_{\mathrm{nfb}}=\frac{E_{\mathrm{nfb}}}{3\left( 1-2\nu_{\mathrm{nfb}} \right)} ,$ | (S2) |
| --- | --- | --- |
|  | $G_{\mathrm{nfb}}=\frac{E_{\mathrm{nfb}}}{2\left( 1+\nu_{\mathrm{nfb}} \right)},$ | (S3) |

where $E_{\mathrm{nfb}}$ (0.16 MPa [1]) is the elastic modulus and $\nu_{\mathrm{nfb}}$ the Poisson’s ratio of the solid non-fibrillar matrix (0.42 [6]).

In the current material model, stress tensor of the fibrillar collagen network arises from the sum of the collagen fiber stresses in each point, including stresses in primary and secondary fibrils [3]. Hence, collagen network stress tensor $\boldsymbol{\sigma}_{\mathrm{fbrl}}$ was defined as

|  | $\boldsymbol{\sigma}_{\mathrm{fb}}= \sum_{k=1}^{totf} {\boldsymbol{\sigma}_{\mathrm{fb}}}^{k},$ | (S4) |
| --- | --- | --- |

where the ${\boldsymbol{\sigma}_{\mathrm{fbrl}}}^{k}$ is the Cauchy stress tensor of the collagen fiber *k* and *totf =* 9 [3] refers to the total number of fibers. Collagen fiber architecture was modeled as observed in young bovine cartilage (50° in the deep zone, 10° in the superficial zone [90° = perpendicular to the surface]) [1]. For each fibril $k$ (primary fibril $k$ = p, secondary fibril $k$ = s), the Cauchy stress tensor ${\boldsymbol{\sigma}_{\mathrm{fb}}}^{k}$ was defined as [3,4]:

|  | ${\boldsymbol{\sigma}_{\mathrm{fb}}}^{k}=\left\{ \begin{matrix} \rho_{z}C\sigma_{\mathrm{fb}}\boldsymbol{e}_{\mathrm{fb}}\otimes\boldsymbol{e}_{\mathrm{fb}}, & \mathrm{if}k=p, \\ \rho_{z}\sigma_{\mathrm{fb}}\boldsymbol{e}_{\mathrm{fb}}\otimes\boldsymbol{e}_{\mathrm{fb}}, & \mathrm{if}k=s, \end{matrix} \right.$ | (S5) |
| --- | --- | --- |

where $\rho_{z}$ is the depth-dependent relative collagen density (see S1 Table ), *C* is the ratio between primary and secondary fibril densities (3.009 [3]), $\boldsymbol{e}_{\mathrm{fb}}$ is the normalized unit vector for fibril orientation [3] and $\otimes$ is the outer product operation. Stress in the collagen fibrils $\sigma_{\mathrm{fb}}$ (scalar) was defined as

|  | $\sigma_{\mathrm{fb}}=\left\{ \begin{matrix} E_{\mathrm{fb}}\varepsilon_{\mathrm{fb}}, & \varepsilon_{f}\geq0, \\ 0, & \varepsilon_{f}<0, \end{matrix} \right.$ | (S6) |
| --- | --- | --- |

where $E_{\mathrm{fb}}$ is the initial constant elastic modulus of a single collagen fiber (20 MPa [1]) and $\varepsilon_{\mathrm{fb}}$ is the logarithmic fibril strain [4].

Fluid flow in the non-fibrillar porous matrix was modeled via Darcy’s law

|  | $q=-k\nabla p ,$ | (S7) |
| --- | --- | --- |

where $q$ is the flow flux in the non-fibrillar matrix, $k$ is the hydraulic permeability ($1.3 \cdot{10}^{-15}m^{4}/(\mathrm{Ns})$ [1]), and $\nabla p$ is the pressure gradient in the cartilage.

The chemical expansion stress caused by repulsion of the negative charge groups in PGs was modeled as

|  | $T_{c}=a_{0}c_{\mathrm{FCD}} \exp\left( -\kappa\frac{\gamma_{\mathrm{ext}}^{\pm}}{\gamma_{\mathrm{int}}^{\pm}}\sqrt{c^{-} \left( c^{-}+c_{\mathrm{FCD}} \right)} \right),$ | (S8) |
| --- | --- | --- |

where $a_{0}$ and $\kappa$ are material constants [3], $\gamma_{\mathrm{ext}}^{\pm}$ and $\gamma_{\mathrm{int}}^{\pm}$ are external and internal activity coefficients [7], and $c^{-}$ is the mobile anion concentration in the cartilage [3,8]. The depth-dependent FCD concentration $c_{\mathrm{FCD}}$ is described as a function of volumetric deformation

|  | $c_{\text{FCD}}=c_{\text{FCD,0}}\frac{n_{\text{f,0}}}{n_{\text{f,0}}-1+J} ,$ | (S9) |
| --- | --- | --- |

where $c_{\text{FCD,0}}$ is the initial depth-wise FCD and $n_{\text{f,0}}$ is the porosity, i.e., fluid volume fraction, both presented in S1 Table.

Donnan osmotic swelling in equilibrium after initial swelling was modeled as

|  | $\Delta\pi=\phi_{\mathrm{int}}RT\left( \sqrt{c_{\mathrm{FCD}}^{2}+4\frac{\left( \gamma_{\mathrm{ext}}^{\pm} \right)^{2}}{\left( \gamma_{\mathrm{int}}^{\pm} \right)^{2}}c_{\mathrm{ext}}^{2}} \right)-2\phi_{\mathrm{ext}}RTc_{\mathrm{ext}},$ | (S10) |
| --- | --- | --- |

where $\phi_{\mathrm{ext}}$ and $\phi_{\mathrm{int}}$ are external and internal osmotic coefficients [7], $c_{\mathrm{ext}}$ is the external salt concentration (0.15 M), $R$ is the molar gas constant (8.3145 J/mol K) and $T$ is the absolute temperature (293 K).

Finally, the total stress tensor $\boldsymbol{\sigma}_{\mathrm{tot}}$ of the cartilage tissue was determined as

|  | $\boldsymbol{\sigma}_{\mathrm{tot}}\boldsymbol{=}\boldsymbol{\sigma}_{\mathrm{nfb}}\mathbf{+}\boldsymbol{\sigma}_{\mathrm{fb}}\mathbf{-}T_{c}\mathbf{I-}\Delta\pi\mathbf{I-}\mu_{f}\mathbf{I},$ | (S11) |
| --- | --- | --- |

where $\mu_{f}$ is the chemical potential of water [5].

**References**

1. Orozco GA, Tanska P, Florea C, Grodzinsky AJ, Korhonen RK. A novel mechanobiological model can predict how physiologically relevant dynamic loading causes proteoglycan loss in mechanically injured articular cartilage. Sci Rep. 2018;8: 1–16. doi:10.1038/s41598-018-33759-3

2. Eskelinen ASA, Mononen ME, Venäläinen MS, Korhonen RK, Tanska P. Maximum shear strain-based algorithm can predict proteoglycan loss in damaged articular cartilage. Biomech Model Mechanobiol. 2019;18: 753–778. doi:10.1007/s10237-018-01113-1

3. Wilson W, Van Donkelaar CC, Van Rietbergen B, Huiskes R. A fibril-reinforced poroviscoelastic swelling model for articular cartilage. J Biomech. 2005;38: 1195–1204. doi:10.1016/j.jbiomech.2004.07.003

4. Wilson W, Van Donkelaar CC, Van Rietbergen B, Ito K, Huiskes R. Stresses in the local collagen network of articular cartilage: A poroviscoelastic fibril-reinforced finite element study. J Biomech. 2004;37: 357–366. doi:10.1016/S0021-9290(03)00267-7

5. Wilson W, Van Donkelaar CC, Huyghe JM. A comparison between mechano-electrochemical and biphasic swelling theories for soft hydrated tissues. J Biomech Eng. 2005;127: 158–165. doi:10.1115/1.1835361

6. Li LP, Buschmann MD, Shirazi-Adl A. A fibril reinforced nonhomogeneous poroelastic model for articular cartilage: Inhomogeneous response in unconfined compression. J Biomech. 2000;33: 1533–1541. doi:10.1016/S0021-9290(00)00153-6

7. Huyghe JM, Houben GB, Drost MR, van Donkelaar CC. An ionised/non-ionised dual porosity model of intervertebral disc tissue. Biomech Model Mechanobiol. 2003;2: 3–19. doi:10.1007/s10237-002-0023-y

8. Huyghe JM, Janssen JD. Quadriphasic mechanics of swelling incompressible porous media. Int J Eng Sci. 1997;35: 793–802. doi:10.1016/s0020-7225(96)00119-x
