# Supplementary material for: Injury-related cell death and proteoglycan loss in articular cartilage: Numerical model combining necrosis, reactive oxygen species, and inflammatory cytokines
Source: PLoS Comput Biol. 2023 Jan 26;19(1):e1010337. doi: 10.1371/journal.pcbi.1010337 (PMC9879441; doi:10.1371/journal.pcbi.1010337)
Supplement: S2 Text — More detailed explanation of the simulations, boundary conditions and finite element mesh of the biomechanical model. (DOCX) [file pcbi.1010337.s002.docx]

**S2 Text. Boundary conditions and finite element mesh used in the biomechanical simulations**

In the biomechanical model, the modeled cartilage explant was subjected to two unconfined compressions following an initial free swelling modeling step. Number of the loading cycles was chosen based on preliminary tests, in which the maximum shear strain distribution at maximum compression amplitude did not change substantially after two cycles. During simulations, we used the following boundary conditions [1]: fluid flow was allowed at the injured and free surfaces while the bottom surface was fixed in the axial direction. No friction was considered between the cartilage and the compressive plate, and the compressive plate was assumed to be impermeable. The finite element mesh used in the biomechanical simulations for the injured cartilage geometry is presented in Fig A.


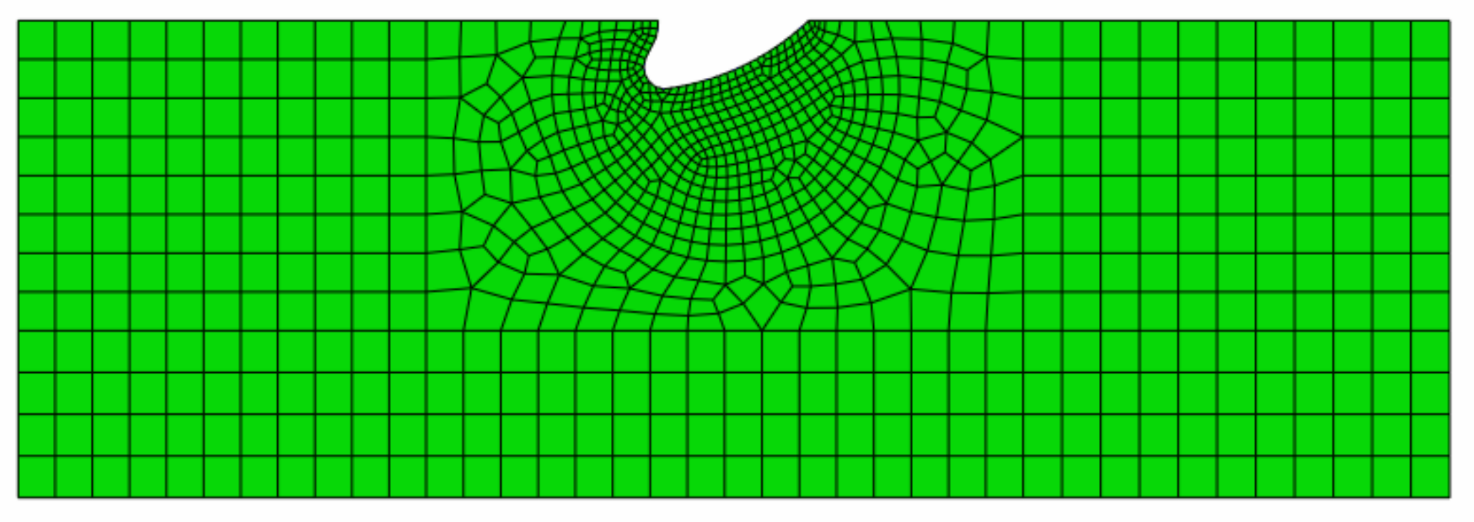


**Fig A. Finite element mesh used in the biomechanical simulations.** Finite element mesh for the injured cartilage geometry including 918 linear axisymmetric elements with pore pressure.

**References**

1. Orozco GA, Tanska P, Florea C, Grodzinsky AJ, Korhonen RK. A novel mechanobiological model can predict how physiologically relevant dynamic loading causes proteoglycan loss in mechanically injured articular cartilage. Sci Rep. 2018;8: 1–16. doi:10.1038/s41598-018-33759-3
