# Supplementary material for: Injury-related cell death and proteoglycan loss in articular cartilage: Numerical model combining necrosis, reactive oxygen species, and inflammatory cytokines
Source: PLoS Comput Biol. 2023 Jan 26;19(1):e1010337. doi: 10.1371/journal.pcbi.1010337 (PMC9879441; doi:10.1371/journal.pcbi.1010337)
Supplement: S3 Text — Biomechanical simulations with higher axial strain amplitude to investigate the initial injury. (DOCX) [file pcbi.1010337.s003.docx]

**S3 Text. Higher axial strain amplitude to investigate the initial impact injury**

Additional biomechanical simulations were conducted to observe how shear strains are distributed during injurious, high-axial-strain loading in the intact cartilage geometry (Fig A). Hence, we conducted a biomechanical simulation with two axial unconfined compressions of 40% axial strain amplitude (1 Hz loading frequency, haversine waveform) and computed the maximum shear strain distribution at the peak strain of the second cycle. The second peak exhibited higher strains compared to the first peak due to fluid flow out of the tissue during the first peak. We used 456 linear axisymmetric elements with pore pressure (element type: CPE4P). Our results showed high maximum shear strains (>90%) distributed in a wide area with peak strains in the center areas of the plug in the superficial zone, where the lesion was placed in the injury geometry.


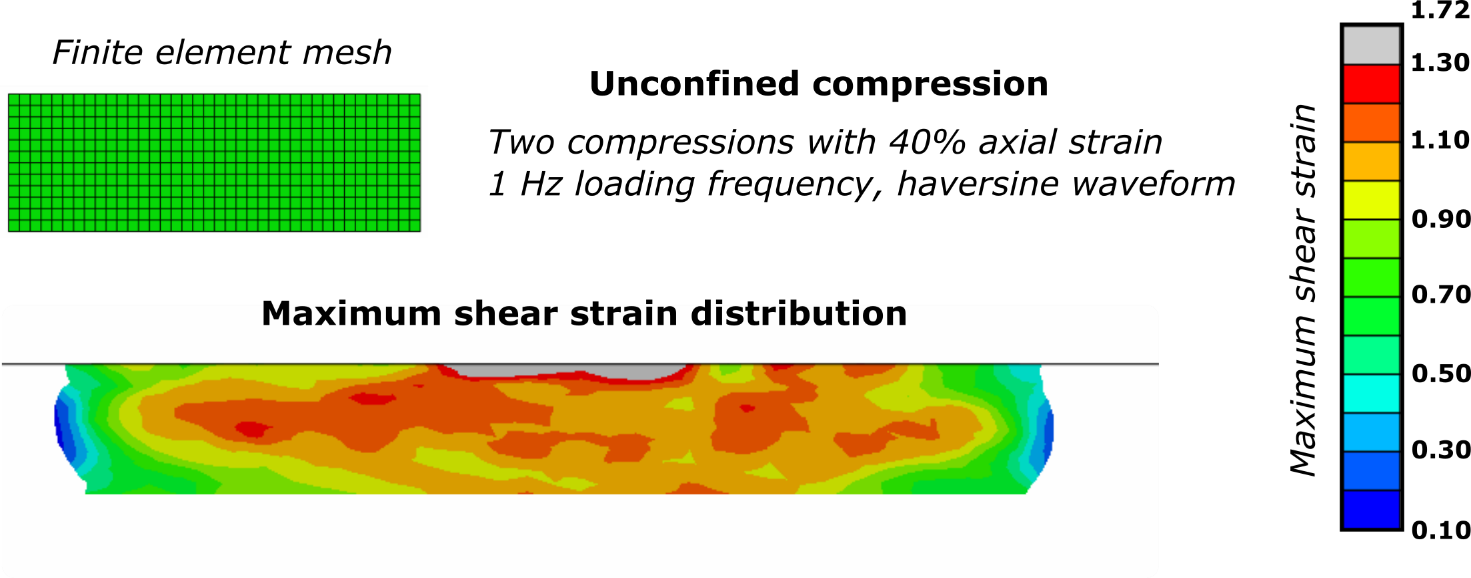


**Fig A. Additional analysis of the higher axial strain to study injurious loading.** Finite element mesh for the intact geometry and the maximum shear strain distributions after unconfined compression with 40% axial strain amplitude. In the injured geometry, the lesion was placed in the areas of high maximum shear strain as shown here.
