# Supplementary material for: Injury-related cell death and proteoglycan loss in articular cartilage: Numerical model combining necrosis, reactive oxygen species, and inflammatory cytokines
Source: PLoS Comput Biol. 2023 Jan 26;19(1):e1010337. doi: 10.1371/journal.pcbi.1010337 (PMC9879441; doi:10.1371/journal.pcbi.1010337)
Supplement: S4 Text — More detailed explanation of the modeling of the proteoglycan loss with the reaction–diffusion equations. (DOCX) [file pcbi.1010337.s004.docx]

**S4 Text. Reaction–diffusion model: simulated changes in proteoglycan content**

The initial PG content (mostly intact aggrecan bound to hyaluronan) was modeled as in the previous work conducted by Kar et al. [1,2]

|  | $C_{\text{PG,0}}=\left( -0.0433z^{4}+0.0431z^{3}-0.0162z^{2}+0.002z+0.0242 \right) \frac{\mathrm{mol}}{m^{3}},$ | (S12) |
| --- | --- | --- |

where *z* is depth normalized with the cartilage thickness so that *z* = 1 at the bottom and *z* = 0 at the explant surface.

Changes in initial PG content were modeled via reaction–diffusion equations including diffusion and chondrocyte-regulated production and degeneration of PGs via interleukin-1 (IL-1) induced aggrecanase production. Since cartilage is a porous structure with small pore size, diffusion of the IL-1 and aggrecanases were modeled using PG concentration-dependent effective diffusion coefficients $D_{\text{eff,}\text{i}}$:

|  | $D_{\text{eff,}\text{i}}=D_{i}\cdot\exp\left( -\lambda_{1,i}\cdot C_{\text{PG}} \right)$, | (S13) |
| --- | --- | --- |

where $D_{i}$ is the diffusivity for species $i$, $\lambda_{1,i}$ is a coefficient defining the relation of the species’ diffusion to the PG concentration $C_{\text{PG}}$ [1].

Degeneration of PGs was controlled via cell death (necrosis and apoptosis) and increased aggrecanase concentration when inhibition of these two factors by decreasing IL-1 concentration led to partial recovery of cartilage PG content. Specifically, the reaction term $R_{\mathrm{PG}}$ in Eq. (1) for the PG was constructed of synthesis/degradation terms $R_{PG, syn}$ and $R_{PG, deg}$:

|  | ${R_{\mathrm{PG}}=R}_{PG, syn}-R_{PG, deg}= {{(C}_{l,c}+C_{d,c})P}_{\mathrm{PG}}\left( 1-0.9z \right)\left( 1-\frac{C_{\mathrm{PG}}}{C_{\mathrm{target}}} \right) - {C_{\mathrm{aga}}k}_{3}\frac{C_{\mathrm{PG}}}{C_{\mathrm{PG}}+K_{m,aga}},$ | (S14) |
| --- | --- | --- |

where $C_{l,c}$ is time-dependent concentration of live cells (decreased by the necrosis, reactive oxygen species and IL-1 induced apoptosis), $C_{d,c}$ is time-dependent concentration of damaged cells (decreased by excessive reactive oxygen species (ROS) and IL-1 induced apoptosis), $P_{\mathrm{PG}}$ is the basal aggrecan production rate [1], $C_{\mathrm{PG}}$ is the PG concentration at time $t$, $C_{\mathrm{target}}$ is the target PG concentration in intact cartilage [1–3], $C_{\mathrm{aga}}$ is the aggrecanase concentration at time *t*, $k_{3}$ is the PG catalytic rate constant [1,4] and $K_{m,aga}$ is the Michaelis constant for aggrecanase [1,4]. Aggrecanase concentration was modeled as $C_{\mathrm{aga}}$:

|  | $\frac{\partial C_{\mathrm{aga}}}{\partial t}=D_{eff,aga}\nabla^{2}C_{\mathrm{aga}}+R_{aga,syn}-R_{aga,deg}=D_{eff,aga}\nabla^{2}C_{\mathrm{aga}}+k_{4}S -k_{5}C_{\mathrm{aga}},$ | (S15) |
| --- | --- | --- |

where $D_{eff,aga}$ is the effective diffusivity of aggrecanase, $k_{4}$ is the stimulus rate coefficient for aggrecanase production [1,5], $S$ is the stimulus term and $k_{5}$ is the aggrecanase proteolytic rate coefficient [1,6]. For more detailed description of the model parameters, readers are referred to Kar et al. [1].

Aggrecanase release from damaged and/or IL-1 signaled chondrocytes was controlled by an aggrecanase stimulus term including the time-delay from intra-cellular responses (e.g., time for mRNA expression and protein translation processes) and protein secretion. In addition to IL-1 induced stimulus $S_{IL-1}$ previously presented by Kar et al. [1], we included a stimulus term $S_{\mathrm{ROS}}$ for ROS induced aggrecanase stimulus [7]. Thus, catabolic chondrocyte stimulus was modeled as a sum of ROS and IL-1 induced stimuli ($S_{IL-1}$ = 0 if only ROS considered; $S_{\mathrm{ROS}}=0$ if only inflammation considered):

|  | $\frac{\partial S}{\partial t}=S_{\mathrm{ROS}}+ S_{IL-1}$  $= \alpha_{\mathrm{ROS}}C_{\mathrm{ROS}}+ \alpha_{IL-1}\left( C^{*}-S \right)$ | (S16) |
| --- | --- | --- |

where $\alpha_{\mathrm{ROS}}$ is the rate constant ($2\cdot{10}^{-10}\frac{1}{s}$) for ROS-induced aggrecanase stimulus, $\alpha_{IL-1}$ is the rate constant for the IL-1-modulated aggrecanase stimulus ($0.4\cdot{10}^{-5}\frac{1}{s}$) [1], and $C^{*}$ is the concentration of IL-1–IL-1-receptor complexes [1]. Since increased ROS production may also sensitize cells to inflammation and induce increased aggrecanase production by further activating intracellular signaling cascades [7,8], $\alpha_{\mathrm{ROS}}$ was defined as a function of the previously used rate constant for IL-1, $\alpha_{IL-1}$ [1], so that $\alpha_{\mathrm{ROS}}=0.00005\alpha_{IL-1}$. In addition, the ROS stimulus constant was visually calibrated by comparing the computationally predicted PG distributions to the experimentally observed optical density in injured cartilage explants and injured explants subjected to cyclic loading [9,10].

**References**

1. Kar S, Smith DW, Gardiner BS, Li Y, Wang Y, Grodzinsky AJ. Modeling IL-1 induced degradation of articular cartilage. Arch Biochem Biophys. 2016;594: 37–53. doi:10.1016/j.abb.2016.02.008

2. Klein TJ, Chaudhry M, Bae WC, Sah RL. Depth-dependent biomechanical and biochemical properties of fetal, newborn, and tissue-engineered articular cartilage. J Biomech. 2007;40: 182–190. doi:10.1016/j.jbiomech.2005.11.002

3. Nagase H, Kashiwagi M. Aggrecanases and cartilage matrix degradation. Arthritis Res Ther. 2003;5: 94–103. doi:10.1186/ar630

4. Wittwer AJ, Hills RL, Keith RH, Munie GE, Arner EC, Anglin CP, et al. Substrate-dependent inhibition kinetics of an active site-directed inhibitor of ADAMTS-4 (aggrecanase 1). Biochemistry. 2007;46: 6393–6401. doi:10.1021/bi7000642

5. Young DA, Lakey RL, Pennington CJ, Jones D, Kevorkian L, Edwards DR, et al. Histone deacetylase inhibitors modulate metalloproteinase gene expression in chondrocytes and block cartilage resorption. Arthritis Res Ther. 2005;7. doi:10.1186/ar1702

6. Yamamoto K, Owen K, Parker AE, Scilabra SD, Dudhia J, Strickland DK, et al. Low density lipoprotein receptor-related protein 1 (LRP1)-mediated endocytic clearance of a disintegrin and metalloproteinase with thrombospondin motifs-4 (ADAMTS-4): Functional differences of non-catalytic domains of ADAMTS-4 and ADAMTS-5 in LRP1 binding. J Biol Chem. 2014;289: 6462–6474. doi:10.1074/jbc.M113.545376

7. Ansari MY, Ahmad N, Voleti S, Wase SJ, Novak K, Haqqi TM. Mitochondrial dysfunction triggers a catabolic response in chondrocytes via ROS-mediated activation of the JNK/AP1 pathway. J Cell Sci. 2020;133. doi:10.1242/jcs.247353

8. Vaamonde-García C, Riveiro-Naveira RR, Valcárcel-Ares MN, Hermida-Carballo L, Blanco FJ, Lõpez-Armada MJ. Mitochondrial dysfunction increases inflammatory responsiveness to cytokines in normal human chondrocytes. Arthritis Rheum. 2012;64: 2927–2936. doi:10.1002/art.34508

9. Eskelinen A, Florea C, Tanska P, Hung H, Frank E, Mikkonen S, et al. Cyclic loading regime considered beneficial does not protect injured and interleukin-1-inflamed cartilage from post-traumatic osteoarhtritis. J Biomech. 2022. Available: https://doi.org/10.1016/j.jbiomech.2022.111181

10. Orozco GA, Tanska P, Florea C, Grodzinsky AJ, Korhonen RK. A novel mechanobiological model can predict how physiologically relevant dynamic loading causes proteoglycan loss in mechanically injured articular cartilage. Sci Rep. 2018;8: 1–16. doi:10.1038/s41598-018-33759-3
