# Supplementary material for: Injury-related cell death and proteoglycan loss in articular cartilage: Numerical model combining necrosis, reactive oxygen species, and inflammatory cytokines
Source: PLoS Comput Biol. 2023 Jan 26;19(1):e1010337. doi: 10.1371/journal.pcbi.1010337 (PMC9879441; doi:10.1371/journal.pcbi.1010337)
Supplement: S5 Text — Mesh sensitivity analysis for the reaction–diffusion model. (DOCX) [file pcbi.1010337.s005.docx]

**S5 Text. Mesh sensitivity**

Mesh density used for the current problem was assured by considering six increasingly dense meshes (Fig A). Denser meshes showed enhanced accuracy in the simulated PG content near the lesion but less changes were observed in the bulk PG content at day 5. Based on the current analysis, a mesh including 2405 triangular elements was chosen for the mechanobiological, time-dependent simulations since more accurate meshes predicted only minor changes in the PG content. Furthermore, currently used triangular mesh provided better convergence for the time-dependent reaction–diffusion simulations than the square element mesh used previously by Orozco et al [1].


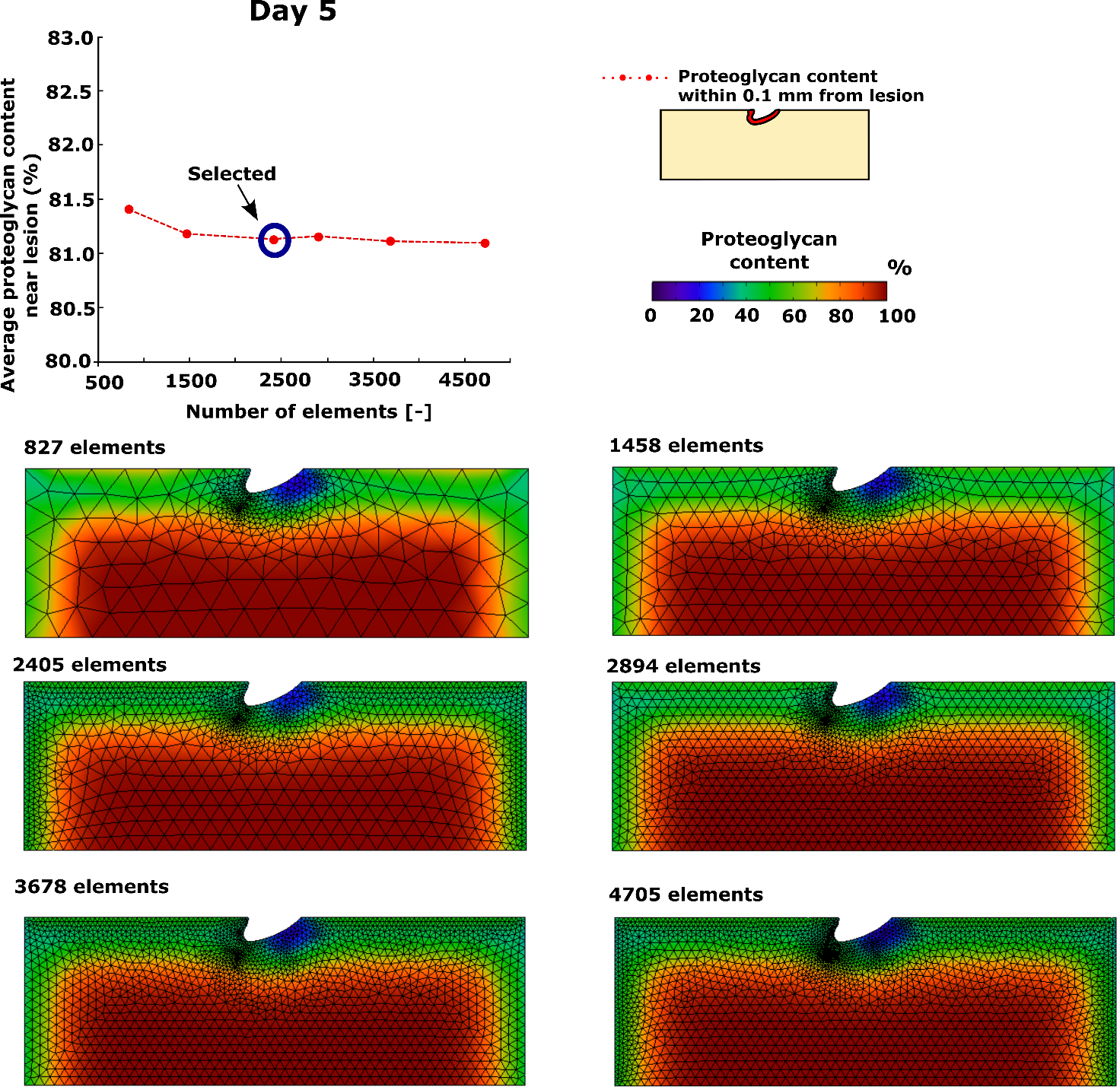


**Fig A. Mesh sensitivity analysis.** Mesh sensitivity analysis for the mechanobiological simulations conducted with the combined model.

**References**

1. Orozco GA, Tanska P, Florea C, Grodzinsky AJ, Korhonen RK. A novel mechanobiological model can predict how physiologically relevant dynamic loading causes proteoglycan loss in mechanically injured articular cartilage. Sci Rep. 2018;8: 1–16. doi:10.1038/s41598-018-33759-3
