# Supplementary material for: Injury-related cell death and proteoglycan loss in articular cartilage: Numerical model combining necrosis, reactive oxygen species, and inflammatory cytokines
Source: PLoS Comput Biol. 2023 Jan 26;19(1):e1010337. doi: 10.1371/journal.pcbi.1010337 (PMC9879441; doi:10.1371/journal.pcbi.1010337)
Supplement: S6 Text — More detailed explanation of the data interpolation from Abaqus to Comsol. (DOCX) [file pcbi.1010337.s006.docx]

**S6 Text. Data interpolation from Abaqus to COMSOL**

To determine an initial condition for the necrotic and damaged cell groups in COMSOL Multiphysics, we constructed cell distributions in MATLAB after biomechanical simulation in ABAQUS. First, we conducted a biomechanical simulation of two dynamic compressions (15% of the initial thickness) of the cartilage plug in ABAQUS [1]. From the biomechanical simulation, we extracted the maximum shear strain values at the finite element integration points (at the maximum compression of the second loading cycle). Next, by using the maximum shear strain distribution and previously used threshold of 50% maximum shear strain [1], we defined locations for necrotic and damaged cells in MATLAB [1]. Similarly, we defined the cell necrosis and cell damage also in the central points and points outside the cartilage geometry (based on nearest integration point value) to assure more accurate cell distributions in the triangular mesh used in COMSOL. Finally, the cell distributions were interpolated into COMSOL via automatic interpolation tool (linear interpolation).

**References**

1. Orozco GA, Tanska P, Florea C, Grodzinsky AJ, Korhonen RK. A novel mechanobiological model can predict how physiologically relevant dynamic loading causes proteoglycan loss in mechanically injured articular cartilage. Sci Rep. 2018;8: 1–16. doi:10.1038/s41598-018-33759-3
