# Supplementary material for: Injury-related cell death and proteoglycan loss in articular cartilage: Numerical model combining necrosis, reactive oxygen species, and inflammatory cytokines
Source: PLoS Comput Biol. 2023 Jan 26;19(1):e1010337. doi: 10.1371/journal.pcbi.1010337 (PMC9879441; doi:10.1371/journal.pcbi.1010337)
Supplement: S1 Table — Variables describing cartilage composition used in the biomechanical material model. (DOCX) [file pcbi.1010337.s007.docx]

**S1 Table.** **Variables describing cartilage composition**.

| **Parameter** | **Value** | **Description** | **Reference** |
| --- | --- | --- | --- |
| **Compositional** |  |  |  |
| $c_{\mathrm{FCD}}$ (mEq/ml) | $-4.4z^{6}+15.2z^{5}-21.0z^{4}+ 14.9z^{3}$  $-5.8z^{2} + 1.1z+ 0.03$ | Depth-wise fixed charge density content | [1] |
| $\rho_{z}$ (-) | $20.6z^{6}-64.4z^{5}+78.1z^{4}- 45.9z^{3}$  $+13.4z^{2} - 1.6z+ 0.96$ | Depth-wise collagen content | [2] |
| $n_{f}$ (-) | $0.85-0.15z$ | Depth-wise fluid fraction | [3] |

Composition of the cartilage in the utilized biomechanical model. Normalized depth $z$ is defined as $z=0$ on the injured surface and $z=1$ on the bottom surface.

**References**

1. Orozco GA, Tanska P, Florea C, Grodzinsky AJ, Korhonen RK. A novel mechanobiological model can predict how physiologically relevant dynamic loading causes proteoglycan loss in mechanically injured articular cartilage. Sci Rep. 2018;8: 1–16. doi:10.1038/s41598-018-33759-3

2. Saarakkala S, Julkunen P. Specificity of fourier transform infrared (FTIR) microspectroscopy to estimate depth-wise proteoglycan content in normal and osteoarthritic human articular cartilage. Cartilage. 2010;1: 262–269. doi:10.1177/1947603510368689

3. Wilson W, Van Donkelaar CC, Van Rietbergen B, Huiskes R. A fibril-reinforced poroviscoelastic swelling model for articular cartilage. J Biomech. 2005;38: 1195–1204. doi:10.1016/j.jbiomech.2004.07.003
