# Supplementary material for: Injury-related cell death and proteoglycan loss in articular cartilage: Numerical model combining necrosis, reactive oxygen species, and inflammatory cytokines
Source: PLoS Comput Biol. 2023 Jan 26;19(1):e1010337. doi: 10.1371/journal.pcbi.1010337 (PMC9879441; doi:10.1371/journal.pcbi.1010337)
Supplement: Attachment — Submitted filename: Response to reviewers.docx [file pcbi.1010337.s008.docx]

**RESPONSE TO REVIEWER CRITIQUE –– PCOMPBIOL-D-22-00983**

We thank the reviewers for their thoughtful reading of our manuscript and the insightful critique they have provided. We were gratified to hear positive support. We have responded point-by-point to specific elements of the critique and correspondingly modified our manuscript. **Changes in the original manuscript are highlighted in yellow.**

**Reviewer #1:**

**(general comment)**

**In this paper, a numerical approach was developed to study the early post-traumatic biomechanical and inflammatory effects on cartilage homeostasis. The results show that cartilage composition can partially recover when post-injury inflammation is mitigated. This is an interesting study, and the paper is well written. The paper can be accepted after some revisions.**

**AUTHOR RESPONSE**

We thank the reviewer for this comment and praise. Please see our point-by-point responses to specific comments below.

**(specific comments)**

**COMMENT 1: Equation (1) shows the reactive transport process of the biochemical species, which normally involves diffusion governed by diffusion coefficient of the species and advection controlled by interstitial fluid flow in cartilage tissue. It is unclear how advection transport was modelled. In addition, the production, binding and degradation process in cartilage is also important. The authors should provide more details by referring to relevant studies in this field (e.g.,** [**https://dx.doi.org/10.3970/mcb.2008.005.133**](https://dx.doi.org/10.3970/mcb.2008.005.133)**).**

**AUTHOR RESPONSE**

Thank you for these excellent points. Currently, our numerical model does not consider the effect of advection of biochemical species, but it would be possible to include in the model with equations similar as Eq. (1) for future studies of this topic.

Indeed, advection due to dynamic loading and associated fluid flow may increase the transport of mobile species (such as IL-1 molecules and free proteoglycan (PG)) into/out of cartilage [1,2]. A previous study conducted by Orozco et al. [3] reported that high fluid velocities were observed near a cartilage lesion during unconfined dynamic compression. Similarly, Zhang et al. [4] reported elevated fluid velocities at distal cartilage regions during repetitive unconfined compression, where advection contributed to the transport of the biomolecules. Hence, advection in injured cartilage might be an important mechanism, a topic which we will consider in our future modeling work.

Production, binding, and degradation processes are also crucial for the understanding of anabolic/catabolic activities in cartilage. These processes can also be implemented with reaction–diffusion equations Eq. (1), utilizing Michaelis–Menten kinetics [1,4,5]. Here, we used Eq. (1) to simulate live cell-dependent PG production [4,6] that was altered by cell death. Cell death was triggered by excessive biomechanical loading and binding of IL-1 to its receptors. The degradation process was controlled by release of aggrecanases in response to necrosis and excessive ROS production as well as IL-1 stimulus.

**AUTHOR ACTION**:

To clarify that advection was not included in the model, we have modified section 2.3 paragraph **Diffusion of aggrecanases and decrease in PG biosynthesis** and added lines 223-224 in the methods section:

*“In the current study, we did not consider fluid flow-induced advective transport of IL-1, aggrecanases, or PG in the Eq. (1).”*

Additionally, we clarified the limitation of excluding advection in lines 562-563 and added more relevant references [1,4] to the manuscript.

We also modified lines 219-221 to help the reader understand the production, binding, and degradation processes considered in the model:

*“The source/sink terms utilized Michaelis–Menten kinetics to model the production and degradation processes (Supplementary material section S4, Eq. (S14)-(S15)) and binding of IL-1 to its receptor like in the model by Kar et al.* [6]*.”*

**COMMENT 2: For necrosis analysis, the current approach is oversimplified. It has been known that cartilage is a fully saturated porous media, composed of interstitial fluid and solid phase (**[**https://doi.org/10.1016/j.triboint.2020.106185**](https://doi.org/10.1016/j.triboint.2020.106185)**), and the consolidation response of cartilage could be significantly changed there is a defect, and ultimately further damage progression (e.g.,** [**https://doi.org/10.1016/j.jmbbm.2017.05.018**](https://doi.org/10.1016/j.jmbbm.2017.05.018)**). The authors should conduct further literature review and provide some in-depth discussions.**

**AUTHOR RESPONSE**

These are good points, and we thank the reviewer for the invitation to discuss them further in the manuscript. The necrosis modeling was clearly a simplification and did not consider changes in consolidation rate [7] or in fluid exchange with the culture medium [8] after several hours of continuous loading. Nevertheless, we consider the current modeling approach still a considerable step forward in mechanistically linking cartilage lesions and necrosis under dynamic loading after traumatic injury.

Our model does not capture the long-term consolidation response possibly present in the experiments during several hours of repetitive loading. However, consolidation may induce time-dependency in tissue deformation, and presumably in peak strain amplitude, during continuous dynamic loading [9]. In addition, fluid exchange with the culture medium [8], decreased boundary resistance [10], and cartilage injuries [7] may decrease the consolidation time leading to decreased fluid pressure and increased local peak strains. As a result, higher local peak strains may broaden the high-strain area from the vicinity of lesions to the deeper layers of the cartilage, causing increased necrosis. Therefore, not considering the continuous loading and consolidation is a clear limitation of our model which might cause underestimation of the predicted necrosis in time.

**AUTHOR ACTION**:

We have clarified the simplifications in our necrosis analysis and provided more discussion about the consolidation response in lines 187-189 and 566-572 with relevant references [7–10]:

Lines 187-189: *“The cartilage injury (lesion) and simplified dynamic loading protocol (two loading*–*unloading cycles in unconfined compression instead of continuous hour-long loading) in the simulations were implemented based on the experiments* [3]*.”*

Lines 587-593: *“After impact-loading and during the continuous dynamic loading, changes in the PG (and collagen) content can also influence the shear strain distributions* [9]*. Moreover, considering fluid exchange with the culture medium* [8]*, cartilage injuries* [7]*, and decreased boundary resistance post-injury* [10] *may result in decreased fluid pressure. This may also broaden high-strain area from the vicinity of lesions to the deeper layers of the cartilage. Hence, due to simplifications in the biomechanical simulations, our model may underestimate the depth-dependent necrosis, apoptosis, and cartilage degeneration.”*

**COMMENT 3: The authors stated that the numerical predications are supported by several previous experimental findings. However, the relevant validation process is missing.**

**AUTHOR RESPONSE**

In this study, we qualitatively compared previous experimental data on cell death and PG content to our numerical predictions. However, a more rigorous validation process would be needed to quantitatively compare between spatiotemporal experimental data and the numerical predictions.

The comparison should include data with quantitative assessment of cell death (necrosis and apoptosis), ROS activity, and PG as well as collagen content. These data should be also acquired with same experimental protocols as simulated by the computational model. We are planning to conduct additional experiments to validate our model predictions and improve the model to match with the chosen experimental protocol. However, our objective in the current study was to identify and localize the injury-related cell-driven cartilage degradation mechanisms and to provide a preliminary numerical model for future calibration. Thus, here we instead focused on estimating the parameters with the best possible data found from the literature.

**AUTHOR ACTION**:

Comparison against the comparative biological data in Fig 2. is discussed in lines 481-482, 506-507 and 524-525. We have clarified the qualitative comparison of the model simulations and the experiments in lines 116-117:

*“We qualitatively compared the simulated cell death and PG content distributions with stained histological cartilage sections from previous in vitro experiments (15,40).”*

In addition, we added more discussion about the limitations of our comparison to the experimental data in lines 594-598:

*“Sixth, although some inflammation and material parameters have been calibrated well* [3,6]*, full model validation is hampered by the small amount of time-dependent biomechanical and biochemical experimental data from different studies. Data for extensive validation should also include fraction of cell death (necrosis and apoptosis), ROS activity, and PG content to further improve the calibration of the related parameters and validate the localized degradation processes induced by mechanisms I-III.”*

**COMMENT 4: In vivo, how is IL-1 transported into cartilage and produced by chondrocytes? Please discuss.**

**AUTHOR RESPONSE**

Thank you for the question. Since there are no blood vessels in cartilage, IL-1 is transported into cartilage mostly through the synovial fluid after production in the surrounding tissues. Alternatively, IL-1 can be produced by the chondrocytes. In an inflamed joint, the major source of inflammatory factors is the synovial membrane. After entering the synovial fluid, inflammatory factors are transported into cartilage via diffusion and advection. Chondrocytes can also produce IL-1 in response to several stimuli, for example, injurious mechanical loading [12] followed by signaling via damage-associated molecular patterns [13], or stimulation of their own IL-1 production in an autocrine manner, triggered by other cytokines such as TNF-α or IL-6 [14]. Although advection and IL-1 production via chondrocytes may be important factors promoting cell death and cartilage degeneration, they were not included in the model simulations for now.

**AUTHOR ACTION**:

We have added the following clarifications about transport of IL-1 into cartilage *in vivo* to the introduction lines 86-91:

*“During the early phases of PTOA, pro-inflammatory cytokines such as interleukin-1 (IL-1), IL-6, IL-18, and tumor necrosis factor-α (TNF-α) originating from the synoviocytes of the synovial lining are secreted to the synovial fluid. These molecules then diffuse and advect into cartilage causing reduced biosynthesis and predisposing the tissue to degeneration that is counter-balanced by anti-inflammatory cytokines (e.g., IL-4, IL-10, IL-13)* [14]*, TIMPs* [15]*, and growth factors (e.g., insulin-like growth factor-1)* [12,14,16]*.”*

In addition, we added discussion how chondrocytes may produce IL-1 in lines 517-519:

*“Lower cell death in our simulated results may be explained by the absence of additional inflammatory mechanisms, such as production of IL-1 by chondrocytes after signaling via DAMPs* [12,13] *or autocrine stimulation via TNF-α* [14]*.”*

Moreover, since IL-1 production by chondrocytes and transport by advection were not included in our model, they are mentioned in limitations in lines 559-563:

*“Here, we have included a few of these mechanisms but, in reality, additional mechanisms may also alter the cartilage degeneration. These mechanisms include:* ***IL-1 production by chondrocytes* [12,14,16]***, IL-1-induced ROS production* [17]*, ROS-induced necrotic cell death* [18,19]*, introduction of MMP-3-driven matrix degradation after injury* [20,21]*,* ***possible fluid flow-dependent PG loss or cytokine transport through cartilage***–***fluid-interfaces via advection* [1,3,4]*,*** *…“*

**REVIEWER #2:**

**(general comment)**

**Reviewer #2: In their manuscript, Kosonen et al. report interesting findings of a new combined numerical model to simulate PTOA-associated pathomechanisms, such as cell death and proteoglycan loss in cartilage. Overall, this seems to be a well-planned project and reliable model.
However, there are some questions and comments, which need to be addressed before considering publication.**

We thank the reviewer 2 for this comment. Please see our point-by-point responses to specific comments below.

**(specific comments)**

**COMMENT 1: In comparison to highly hypocellular human cartilage, bovine or porcine cartilage is rather cell-rich. This, of course, also influences the biomechanical properties of the tissue. The numerical model seems to be mainly based on preliminary results from a bovine injury model. In the literature, there are also various reports focusing on the cellular response (cell death and cartilage degeneration) after ex vivo cartilage trauma using human cartilage. The authors should refer to human models and discuss the similarities or differences.**

**AUTHOR RESPONSE**

This is an excellent point from the reviewer. Specifically, young bovine cartilage has higher cell concentration than adult human cartilage [22,23]. In addition, depending on many factors, such as age and degree of osteoarthritis, bovine and human cartilage may have different structure and biomechanical properties [24–26]. Lower cellularity and different biomechanical properties in human *in vitro* studies most likely affect the downstream cellular responses. However, evidence suggests [16,27,28] that similar mechanisms contribute to cell death and cartilage degradation in human and bovine models after traumatic injury.

Biomechanically and biochemically induced apoptosis, necrosis, and cartilage degeneration have been observed in several animal and human *in vitro* models. As in bovine/porcine injury-models, *in vitro* human models have reported necrosis and subsequent apoptosis after injury [29–32]. For example, Phillips et al. [32] and Riegger et al. [27,28] reported 40% cell death in injuriously loaded bovine cartilage (25 MPa impact in unconfined compression) and human cartilage (0.59 J single-impact loading) 1 day after injury, respectively. Both suggested that decreased cell viability may be due to apoptosis and necrosis. In addition, human and bovine injury-models have shown regional cell death near lesions, whereas it was reported to be less extensive in hypocellular human cartilage [25]. Moreover, injuries and inflammation can cause highly increased expression/activity of matrix-destructing enzymes and PG loss in both animal and human models [27,33,34]. Therefore, cell-driven cartilage degeneration may be dampened in human cartilage due to lower cellularity when compared to animal cartilage, but it likely still plays an important role in the acute and subacute degradation processes in both models.

**AUTHOR ACTION**:

We have added references to the human cartilage models and more discussion about the comparison between human/animal *in vitro* models and the simulated results of necrosis, ROS induced apoptosis, and PG degradation in lines 456-461, 500-503, and 528-530.

Lines 456-461: *“Similarly, human in vitro impact-models have also shown necrotic cell death occurring near lesions and increased catabolic expression in cartilage after injury* [25,28]*. Albeit we did not consider necrosis caused by the initial impact injury, our model could capture the necrotic cell death near the lesion due to high strains resulting from dynamic loading of injured geometry as well as the following rapid PG degeneration due to high catabolic activity in these areas.”*

Lines 500-503: *”A similar interplay between damaged cells and increased ROS production leading to cell death and PG loss has been observed experimentally in animal models* [35–38] *and also in human cartilage explants* [27]*.”*

Lines 528-530 *“Previous experiments have shown that injuries can potentiate inflammation causing highly increased expression/activity of matrix-destructing enzymes and PG loss in bovine and human explants* [34]*.”*

**COMMENT 2: The advantage of this numerical model remains unclear. It seems to “predict” or rather reproduce the findings of preliminary ex vivo cartilage injury models. What exactly is the benefit? Might it be possible to replace in vitro or even in vivo testing? Although, an ex vivo cartilage injury model is already a simplified model: Is it really possible to break down this complex system, including cell-matrix interaction, different cell types (there is not only one type of chondrocytes!), release of various cytokines, chemokines, catabolic enzymes and so on, to a few parameters to predict a potential outcome?**

**AUTHOR RESPONSE**

Thank you for the question. The computational model can pinpoint the underlying degradation mechanisms leading to the measured result. These mechanisms are difficult or impossible to study otherwise (i.e., experimentally). The benefit is enhanced understanding of *in vitro* observations via numerical simulations focusing on a few of the mechanisms considered most important for local cell death and PG loss. Our model adds to the understanding of how biomechanical loading and inflammation trigger cell-level responses, for example necrosis, oxidative stress, and catabolic cell reactions, resulting in compositional changes in cartilage as observed experimentally after injury.

The current model does not aim to replace *in vitro*/*in vivo* testing. In fact, more quantitative experimental evidence is needed to calibrate the model and further elucidate how different mechanisms affect cartilage degradation after injury. Nevertheless, after proper calibration, the model may also have potential to replace testing of different *in vitro* protocols to enhance the development of tissue engineered cartilage. Furthermore, the long-term goal is to predict osteoarthritis progression in clinical applications and evaluate virtually the effect of treatments, related to alterations in the biomechanical (e.g., rehabilitation) and biochemical (e.g., pharmaceutical treatment) environment. This kind of *in silico* modeling framework could help in clinical decision making on the suitable treatment strategy. Since capturing every biochemical aspect of the degradation process is likely untenable, simplifications must be adopted to analyze the net effect of each biomarker on the degradation. Thus, we consider pinpointing and implementing the most influential mechanisms into degradation modeling frameworks which would bring the most value to healthcare.

**AUTHOR ACTION**:

We have added lines 104-105 and 105-108 to the introduction and modified/added lines 434-435, 557-560, and 611-617 in the discussion to clarify the benefit of the current modeling framework and to highlight that the model simulated the net effect of different mechanisms rather than separately considered multiple complex mechanisms.

Lines 104-105: *“Yet, there are no numerical approaches that would model both trauma-related cellular mechanisms and subsequent changes in cartilage composition.”*

Lines 106-108: *“Thus, we aimed to develop a new 2-D cell-and-tissue-level mechanobiological model of cartilage degeneration* [3,6,39] *to localize and predict injury- and inflammation-related chondrocyte responses and subsequent early-stage cartilage degeneration.”*

Lines 434-435: *“Comparison of the model simulations and biological data implies that our model can pinpoint relevant underlying degradation mechanisms leading to the cartilage degeneration.”*

Lines 557-560: *“All mechanisms inducing cartilage degradation are nearly impossible to include in a computational model, but we believe cartilage degeneration can be predicted by estimating the net effect of the most significant mechanisms. Here, we have included a few of these mechanisms but, in reality, additional mechanisms may also alter the cartilage degeneration. These mechanisms may include: …”*

Lines 611-617: *“One potential example could be merging this framework with musculoskeletal–finite element modeling workflow* [40–42] *which considers patient-specific joint geometry and loading. When adding inflammatory biomarkers from the synovial fluid, and possibly the defect size, to the workflow with our new cell-level mechanobiological model, virtual evaluation of the effects of biomechanical (such as rehabilitation) and biochemical (for example pharmaceutical treatment) interventions on cartilage health would become possible. This would enable optimizing the treatment and help in clinical decision making.”*

**COMMENT 3: Results part – In generally, the impact energy and type (repetitive/ cyclic or single impact) should be described in more detail in the results. For example, it is mentioned that “… we did not model the injurious loading itself, but rather the subsequent physiologically relevant dynamic loading of injured cartilage”. The reader should understand the results without searching the missing information in the Materials part.**

**AUTHOR RESPONSE**

Thank you for raising this point. We have clarified the issue in Results part.

In short, we did not model the injurious loading *per se*. However, the lesion in the model geometry was observed experimentally after a single compression of 50% axial strain (i.e., 0.5 mm) with strain rate of 100%/s in unconfined compression as presented in line 172. The impact energy was not measured. The subsequent compressive dynamic loading was the same as in experiments: 15% strain amplitude, 1 Hz haversine waveform in unconfined compression, as said on line 173. The resulting strains after two simulated cycles were used to trigger the cell responses and downstream tissue degradation.

**AUTHOR ACTION**:

Added to the results, lines 331-332: *“Simulated necrosis was localized near the cartilage lesion (Fig. 1A) after dynamic loading (initial impact-loading was not modeled).”*

**COMMENT 4: The authors simulated cell death (apoptosis/necrosis) at day 5. For example, line 335, “… average cell apoptosis of 6.5% …” What exactly does the 6.5 % represent? Apoptosis starts after injury and continues until day 5. Does the 6.5 % refers to the cumulative number of death cells or the apoptosis rate at day 5 (without considering the cells, which underwent apoptosis before)?**

**AUTHOR RESPONSE**

Thank you for the question, this was indeed unclear. Throughout the manuscript, the cell death percentages convey the cumulative fraction of dead cells on the specific day. Fraction of the dead cells was defined as the dead cells on the specified day and area. We studied an area near the lesion (0.1 mm from lesion edge showing most cell death [3]) and the whole cartilage geometry. In both areas, the percentual amount of live/dead cells were computed by dividing the integrated cell count over the area at specific day by the count in the area at day 0.

**AUTHOR ACTION**:

We have modified lines 333, 355, 393 and 404 to clarify that the expression is meant to convey the cumulative fraction of dead cells.

**COMMENT 5: Why did the authors focus on IL-1b as pro-apoptotic cytokine? TNF is usually the predominant pro-apoptotic/pro-necroptotic cytokine. Is it because TNF is rather released by synovial cells than chondrocytes? Speaking of which, the authors have not included synovial inflammation but only chondrocyte-derived cytokine. If this is correct, the authors should discuss why they did not include TNF/ synovial inflammation, because this would represent the in vivo situation.**

**AUTHOR RESPONSE**

We thank the reviewer for this important insight. We focused on the net effect of IL-1 cytokines since they are well-known triggers of apoptosis [18] and they are present in the early, acute tissue responses after injury also in the synovial inflammation [43]. Moreover, IL-1 was used in the previous experiments [44]. We acknowledge that TNF is considered as an another important cytokine causing extensive cell death [45], certainly of interest to be included in the future model developments. Here, we did not have access to TNF diffusion coefficient in young bovine cartilage and TNF biochemical degradation coefficients, which further guided us to focus only on IL-1 for now.

**AUTHOR ACTION**:

We have added lines 572-576 to justify the selection of IL-1 for the simulations:

*“In the human joint, also other pro-inflammatory cytokines such as TNF-*$\alpha$*, regulate apoptosis* [46,47]*. Their effects would be possible to include into the model with Eq. (1). However, for now, we did not incorporate TNF-*$\alpha$*, since we had no information about its diffusion/reaction coefficients, and it was not used in the experiments utilized for model comparison.”*

**COMMENT 6: Discussion – Line 415: “The injury can also sensitize live cells to turn catabolic more easily by later mechanical and inflammatory signals causing more extensive cell death if catabolic signals are not ceased.” This sentence is very complex and hard to understand. Rephrasing is required.**

**AUTHOR RESPONSE**

Thank you, we have rephrased the sentence.

**AUTHOR ACTION**:

We have added the following on lines 439-441:

*“**The injury can also stimulate catabolic pathways in live cells and predispose them to further harmful mechanical and inflammatory stimuli. As a result of high susceptibility to harmful stimuli, they may die or express catabolic enzymes easier than undamaged cells.”*

**COMMENT 7: Line 418 ff: besides apoptosis, the authors discuss necrotic cell death after cartilage injury. There is also a report about necroptosis (regulated necrosis) after blunt mechanical impact, which describes the subsequent release of DAMPs, triggering pro-inflammatory processes.**

**AUTHOR RESPONSE**

We thank the reviewer for this insight. We have added a sentence to discussion.

**AUTHOR ACTION**:

Since necroptosis could be also an important form of cell death promoting cartilage degradation, we have added lines 447-450 to the discussion:

*“In addition to necrosis, necroptosis (regulated necrosis) can be involved in the release of DAMPs after injury stimulating the inflammatory response in chondrocytes* [48] *and, thus, participate in accelerated degradation of cartilage post-injury* [49]*.”*

**COMMENT 8: Line 433: “In our simulated necrosis model, aggrecanases were released only at day 0 in response to cell necrosis near the lesion (Fig. 4A).” Could the authors please clarify this statement? Why should aggrecanases be release in response to necrosis (at day 0)? It has previously been demonstrated that expression of some aggrecanases and MMPs is only slightly induced or even downregulated during the acute phase (0-24 h) and peaks a few days after ex vivo trauma (3 – 7 days). This might be linked to oxidative stress and subsequent induction of redox-sensitive pathways (p38,NFkB).**

**AUTHOR RESPONSE**

We appreciate these truly relevant questions from the reviewer. Several authors have reported near-lesion necrosis [32,50], aggrecanase release [51,52], and GAG loss [53] as an early response to injurious loading.

Increased amount of necrotic cells [32] and expression of aggrecanases have been reported 24 hours post-injury [33]. Although no significant effect was previously reported to the bulk PG loss (GAG release) after inhibition of aggrecanase activity in injured cartilage [54], a recent study [51] reported locally concentrated aggrecanase activity and GAG degradation in injured cartilage after a 24-hour culture. This suggests aggrecanases could contribute to the early and locally concentrated degeneration of cartilage. These aggrecanases could be newly synthesized or originate from the natural tissue reservoirs [55]. Here, we modeled the net effect of injury on necrosis, mostly occurring locally near the lesion in the first hours of day 0 in accordance with the current literature. Nevertheless, we did not specify the release/activation of catabolic enzymes. Necrotic cells show ruptured cell membrane, thus possibly allowing release of activated aggrecanases from the cytosol to the local extracellular matrix [52,55,56]. With these assumption, the model predicted similar results as observed in previous experiments; rapid release of aggrecanases near the lesion [51], in areas experiencing necrosis [32].

Moreover, we included the subacute activation of catabolic intracellular pathways through a stimulus term (presented in Supplementary material Eq. (S16)) and release of aggrecanases associated with local oxidative stress/ROS activity with Eq. (1) [27]. We recognize that the time delay of a few hours in the enzyme secretion could be another important parameter to consider in short simulations, but such change would have negligible effect on the PG loss in our 30-day simulations. This time-dependency would need a whole new set of experiments to fully validate the model.

**AUTHOR ACTION**:

We have clarified our decision of choosing the necrosis release at day 0 in lines 236-239 and 465-470. We also provided additional references [51,55] to support our choice.

Lines 465-470: *“The predicted early burst of enzymatic activity is supported by experiments showing increased aggrecanase expression in injured bovine cartilage 1 day after experimental injury* [33]*. In addition, Merrild et al.* [51] *reported localized aggrecanase activity near the lesion 1 day after cartilage injury, which could represent newly synthesized aggrecanase or that originating from the tissue reservoirs* [54]*. Since aggrecanases may be activated intracellularly* [55]*, they may be capable of degrading ECM PGs when released after cell membare rupture in necrosis.”*

We also clarified the reason to separate acute necrosis and the following subacute PG degradation in the model in lines 476-484:

*“Interestingly, our results from necrosis-only model suggest that at the local level cartilage can partially recover its PG content and reach a steady state in 12 days. This implies that after acute PG degradation and loss, a decrease in aggrecanase concentration and diffusion of PGs from deeper layers of the cartilage can promote PG recovery.* *It has been suggested that early and localized PG degeneration may be a part of the repair process of minor cartilage injuries* [51]*, but continued expression of catabolic enzymes may lead to pathological degradation. However, in previous experiments* [3,11]*, low PG content was still observed near the lesion at day 12. This suggests that,* ***in addition to immediate necrosis, further subacute mechanisms associated with cell damage (e.g., oxidative stress and ROS overproduction) should be included in the simulations to better detect the temporal changes in injured cartilage****.”*

**COMMENT 9: Line 499: The “partial recovery” observed after decrease of IL-1b should be discussed with/ compared to the literature. Why is there an increase of anabolism, which cells contribute to the regeneration of the tissue, … is this recovery induced by the “subsequent physiologically relevant dynamic loading of injured cartilage”? The influence on physiological dynamic loading on cartilage metabolism, in particular anabolism, should be included in the discussion.**

**AUTHOR RESPONSE**

We thank the reviewer for the questions. When IL-1 concentration was decreased as described in Eq. (8) (see also Fig. 8A), our model suggests that the increased anabolism in cartilage is achieved by rescued PG synthesis (lower apoptosis) and decreased aggrecanase production (see Eq. (1) and Eq. (S14) in Supplementary material section S4). These together enabled partial recovery of the PG content. The current model was not implemented with the beneficial effects of the physiological loading, but we added this important aspect to the discussion.

Partial recovery of the cartilage PG content could be possible after traumatic joint injury, as suggested by an earlier study about rabbit anterior cruciate ligament transection [57]. Although loading of injured cartilage may induce cell damage and death, our model suggested that fast removal of IL-1 may still result in partial PG recovery driven by healthy cells. Specifically, our numerical model predicted PG recovery between days 10 and 30 (Fig. 8E, purple line) after removal of most IL-1 from the culture medium (day 10 in Fig. 8A, purple line). In a similar time-window, previous *in vitro* experiments showed partial recovery of uninjured bovine cartilage GAG content over 3 weeks after changing the culture medium into medium that does not have IL-1 [58]. In addition, the most severely degraded regions in the uninjured but inflamed cartilage samples showed less PG recovery compared to areas experiencing less degeneration. Similarly, our model predictions showed less recovery in highly degenerated areas near the lesion (Fig. 8G). Therefore, our model could provide reasonable predictions of the cartilage PG content recovery.

Physiologic dynamic loading also plays a major role in cartilage metabolism. For instance, previous studies have shown that physiologic dynamic loading can alleviate the catabolic effects of IL-1 [59] and especially increase the synthesis of the ECM matrix in live cells [11]. Although being beneficial during 2–3 days post-injury, a recent study [11] also suggested physiological loading alone was not able to rescue injured-and-inflamed cartilage from cartilage degeneration in a 12-day culture. Hence, anti-inflammatory treatment is also most likely needed to inhibit inflammation induced catabolism and initiate the recovery.

**AUTHOR ACTION**:

We have clarified and added more discussion of the recovery mechanisms and comparison of modeling results against literature [57,58] in lines 539-551. To acknowledge that ignoring beneficial effects of dynamic loading clearly is a limitation of our model, we added lines 565-567.

Lines 539-551: *“The result of possible partial recovery suggests that inhibition of cytokine activity or rapid cytokine clearance from the culture medium/joint space could suppress production of proteolytic enzymes and rescue PG synthesis via reduced cell death (see Eq. (1) and Eq. (S14) in the Supplementary material section S1.4). Similar PG recovery has also been reported in experimental studies* [57,58]*.* *Specifically*, *our numerical model predicted PG recovery between days 10 and 30 (Fig 8E, purple line) after the removal of most IL-1 from the culture medium (day 10 in Fig. 8A, purple line). During a similar time-window as shown by our model, previous in vitro experiments showed partial recovery of uninjured bovine cartilage GAG content over 3 weeks after changing the culture medium to that without IL-1* [58]*. Interestingly, less recovery was observed in the most severely degraded regions compared to the more intact regions of the cartilage after IL-1 treatment. Similarly, our model predicted less recovery in highly degenerated areas near the lesion compared to the more intact areas away from lesion (Fig. 8G). These findings indicate that our model provided reasonable predictions of the cartilage recovery and adaptation.”*

Lines 565-567: *“Moreover, our model does not consider the beneficial effects of moderate dynamic loading, such as alleviation of the catabolic effects of IL-1 and, especially, increased synthesis of the ECM matrix components in live cells* [11,59,60]*.”*

**COMMENT 10: Line 543 ff: The future outlook to “produce patient-specific predictions of early cartilage degeneration” is not clear. How exactly should that work? And what exactly does “patient-specific” mean in this context? Which parameter would be necessary to make predictions (e.g. synovial inflammation, defect size, weight, …)??**

**AUTHOR RESPONSE**

Our outlook certainly needs clarification. We vision that we can utilize the knowledge from the tissue level to implement the degenerative mechanisms into patient-specific joint-level finite element models to predict osteoarthritis progression [42,61]. For this aim, understanding cartilage degradation through experiments and computational modeling in the tissue level (our current study) is highly important. Patient-specific models may include joint geometry (e.g., via MRI or X-ray), inflammatory molecule concentrations in synovial fluid, and gait analysis to obtain loading conditions in the joint. Development of such musculoskeletal–finite element modeling workflow has already begun [40–42]. When such a workflow is enhanced with cartilage degradation models in the cell–tissue-level, a calibrated workflow could be used to estimate patient-specific cartilage degradation and possible intervention strategies. Model predictions could then help in choosing personalized intervention to mitigate PTOA progression.

**AUTHOR ACTION**:

We have clarified our future outlook and provided additional references [40–42] to enlighten possible future patient-specific modeling frameworks in lines 604-621:

*“In the future, multiscale mechanobiological models may be feasible to produce patient-specific predictions of early cartilage degeneration. Future developments of the model could include the implementation of the pericellular matrix to investigate the mechano-inflammatory crosstalk between the pericellular matrix and the chondrocyte at the early phases of cartilage injury* [62,63]*. In addition, extensive experimental research is still needed to elucidate the injury-related mechanotransduction pathways, cell death, and ROS kinetics, which could provide time-dependent quantitative data to calibrate and enhance current modeling framework. Thus, we are planning to conduct additional experiments to validate our tissue-level model predictions which could then provide validated improvements in the current joint-level models* [42,61]*. One potential example could be merging this framework with musculoskeletal–finite element modeling workflow* [40–42] *which considers patient-specific joint geometry and loading. When adding inflammatory biomarkers from the synovial fluid, and possibly the defect size, to the workflow with our new cell-level mechanobiological model, virtual evaluation of the effects of biomechanical (such as rehabilitation) and biochemical (for example pharmaceutical treatment) interventions on cartilage health would become possible. This would enable optimizing the treatment and help in clinical decision making. Our current model already considers ROS (Eqs. (3)-(6)) and could provide means to simulate, for example, the effect of N-acetylcysteine treatment on oxidative stress, ROS production, cell death, and cartilage health* [64]*. Eventually, the model predictions could produce cost-efficient optimized intervention strategies, to mitigate early cartilage degeneration.”*

**Additional References Supporting this Response**

1.	Zhang L, Gardiner BS, Smith DW, Pivonka P, Grodzinsky A. The effect of cyclic deformation and solute binding on solute transport in cartilage. Arch Biochem Biophys. 2007;457: 47–56. doi:10.1016/j.abb.2006.10.007

2.	Gardiner B, Smith D, Pivonka P, Grodzinsky A, Frank E, Zhang L. Solute transport in cartilage undergoing cyclic deformation. Comput Methods Biomech Biomed Engin. 2007;10: 265–278. doi:10.1080/10255840701309163

3.	Orozco GA, Tanska P, Florea C, Grodzinsky AJ, Korhonen RK. A novel mechanobiological model can predict how physiologically relevant dynamic loading causes proteoglycan loss in mechanically injured articular cartilage. Sci Rep. 2018;8: 1–16. doi:10.1038/s41598-018-33759-3

4.	Zhang L, Gardiner BS, Smith DW, Pivonka P, Grodzinsky A. A fully coupled poroelastic reactive-transport model of cartilage. MCB Mol Cell Biomech. 2008;5: 133–153.

5.	Kar S, Smith DW, Gardiner BS, Grodzinsky AJ. Systems based study of the therapeutic potential of small charged molecules for the inhibition of IL-1 mediated cartilage degradation. PLoS One. 2016;11: 1–38. doi:10.1371/journal.pone.0168047

6.	Kar S, Smith DW, Gardiner BS, Li Y, Wang Y, Grodzinsky AJ. Modeling IL-1 induced degradation of articular cartilage. Arch Biochem Biophys. 2016;594: 37–53. doi:10.1016/j.abb.2016.02.008

7.	Miramini S, Smith DW, Zhang L, Gardiner BS. Journal of the Mechanical Behavior of Biomedical Materials The spatio-temporal mechanical environment of healthy and injured human cartilage during sustained activity and its role in cartilage damage. J Mech Behav Biomed Mater. 2017;74: 1–10. doi:10.1016/j.jmbbm.2017.05.018

8.	Liao JJ, Smith DW, Miramini S, Gardiner BS, Zhang L. A coupled contact model of cartilage lubrication in the mixed-mode regime under static compression. Tribol Int. 2020;145: 106185. doi:10.1016/j.triboint.2020.106185

9.	Zhang L, Miramini S, Smith DW, Gardiner BS, Grodzinsky AJ. Time Evolution of Deformation in a Human Cartilage Under Cyclic Loading. Ann Biomed Eng. 2015;43: 1166–1177. doi:10.1007/s10439-014-1164-8

10.	Woodhouse FG, Gardiner BS, Smith DW. Short-term consolidation of articular cartilage in the long-term context of osteoarthritis. J Theor Biol. 2015;368: 102–112. doi:10.1016/j.jtbi.2015.01.003

11.	Eskelinen A, Florea C, Tanska P, Hung H, Frank E, Mikkonen S, et al. Cyclic loading regime considered beneficial does not protect injured and interleukin-1-inflamed cartilage from post-traumatic osteoarhtritis. J Biomech. 2022. Available: https://doi.org/10.1016/j.jbiomech.2022.111181

12.	Lieberthal J, Sambamurthy N, Scanzello CR. Inflammation in joint injury and post-traumatic osteoarthritis. Osteoarthr Cartil. 2015;23: 1825–1834. doi:10.1016/j.joca.2015.08.015

13.	Rosenberg JH, Rai V, Dilisio MF, Agrawal DK. Damage-associated molecular patterns in the pathogenesis of osteoarthritis: potentially novel therapeutic targets. Mol Cell Biochem. 2017;434: 171–179. doi:10.1007/s11010-017-3047-4

14.	Wojdasiewicz P, Poniatowski ŁA, Szukiewicz D. The role of inflammatory and anti-inflammatory cytokines in the pathogenesis of osteoarthritis. Mediators Inflamm. 2014;2014. doi:10.1155/2014/561459

15.	Yamamoto K, Wilkinson D, Bou-Gharios G. Targeting Dysregulation of Metalloproteinase Activity in Osteoarthritis. Calcif Tissue Int. 2021;109: 277–290. doi:10.1007/s00223-020-00739-7

16.	Li Y, Wang Y, Chubinskaya S, Schoeberl B, Florine E, Kopesky P, et al. Effects of insulin-like growth factor-1 and dexamethasone on cytokine-challenged cartilage: Relevance to post-traumatic osteoarthritis. Osteoarthr Cartil. 2015;23: 266–274. doi:10.1016/j.joca.2014.11.006

17.	Bolduc JA, Collins JA, Loeser RF. Reactive oxygen species, aging and articular cartilage homeostasis. Free Radic Biol Med. 2019;132: 73–82. doi:10.1016/j.freeradbiomed.2018.08.038

18.	Charlier E, Relic B, Deroyer C, Malaise O, Neuville S, Collée J, et al. Insights on molecular mechanisms of chondrocytes death in osteoarthritis. Int J Mol Sci. 2016;17. doi:10.3390/ijms17122146

19.	Blanco FJ, Ochs RL, Schwarz H, Lotz M. Chondrocyte apoptosis induced by nitric oxide. Am J Pathol. 1995;146: 75–85.

20.	Patwari P, Cook MN, DiMicco MA, Blake SM, James IE, Kumar S, et al. Proteoglycan degradation after injurious compression of bovine and human articular cartilage in vitro: Interaction with exogenous cytokines. Arthritis Rheum. 2003;48: 1292–1301. doi:10.1002/art.10892

21.	Lin PM, Chen CTC, Torzilli PA. Increased stromelysin-1 (MMP-3), proteoglycan degradation (3B3- and 7D4) and collagen damage in cyclically load-injured articular cartilage. Osteoarthr Cartil. 2004;12: 485–496. doi:10.1016/j.joca.2004.02.012

22.	Jadin KD, Wong BL, Bae WC, Li KW, Williamson AK, Schumacher BL, et al. Depth-varying density and organization of chondrocytes in immature and mature bovine articular cartilage assessed by 3D imaging and analysis. J Histochem Cytochem. 2005;53: 1109–1119. doi:10.1369/jhc.4A6511.2005

23.	Lotz M, Loeser RF. Effects of aging on articular cartilage homeostasis. Bone. 2012;51: 241–248. doi:10.1016/j.bone.2012.03.023

24.	Ebrahimi M, Finnilä MAJ, Turkiewicz A, Englund M, Saarakkala S, Korhonen RK, et al. Elastic, Dynamic Viscoelastic and Model-Derived Fibril-Reinforced Poroelastic Mechanical Properties of Normal and Osteoarthritic Human Femoral Condyle Cartilage. Ann Biomed Eng. 2021;49: 2622–2634. doi:10.1007/s10439-021-02838-4

25.	Démarteau O, Pillet L, Inaebnit A, Borens O, Quinn TM. Biomechanical characterization and in vitro mechanical injury of elderly human femoral head cartilage: comparison to adult bovine humeral head cartilage. Osteoarthr Cartil. 2006;14: 589–596. doi:10.1016/j.joca.2005.12.011

26.	Jeffrey JE, Aspden RM. The biophysical effects of a single impact load on human and bovine articular cartilage. Proc Inst Mech Eng Part H J Eng Med. 2006;220: 677–686. doi:10.1243/09544119JEIM31

27.	Riegger J, Joos H, Palm HG, Friemert B, Reichel H, Ignatius A, et al. Antioxidative therapy in an ex vivo human cartilage trauma-model: attenuation of trauma-induced cell loss and ECM-destructive enzymes by N-acetyl cysteine. Osteoarthr Cartil. 2016;24: 2171–2180. doi:10.1016/j.joca.2016.07.019

28.	Riegger J, Joos H, Palm HG, Friemert B, Reichel H, Ignatius A, et al. Striking a new path in reducing cartilage breakdown: combination of antioxidative therapy and chondroanabolic stimulation after blunt cartilage trauma. J Cell Mol Med. 2018;22: 77–88. doi:10.1111/jcmm.13295

29.	Pascual Garrido C, Hakimiyan AA, Rappoport L, Oegema TR, Wimmer MA, Chubinskaya S. Anti-apoptotic treatments prevent cartilage degradation after acute trauma to human ankle cartilage. Osteoarthr Cartil. 2009;17: 1244–1251. doi:10.1016/j.joca.2009.03.007

30.	Chen CT, Burton-Wurster N, Borden C, Hueffer K, Bloom SE, Lust G. Chondrocyte necrosis and apoptosis in impact damaged articular cartilage. J Orthop Res. 2001;19: 703–711. doi:10.1016/S0736-0266(00)00066-8

31.	Dlima DD, Hashimoto S, Chen PC, Colwell CW, Lotz MK. Human chondrocyte apoptosis in response to mechanical injury. Osteoarthr Cartil. 2001;9: 712–719. doi:10.1053/joca.2001.0468

32.	Phillips DM, Haut RC. The use of a non-ionic surfactant (P188) to save chondrocytes from necrosis following impact loading of chondral explants. J Orthop Res. 2004;22: 1135–1142. doi:10.1016/j.orthres.2004.02.002

33.	Lee JH, Fitzgerald JB, DiMicco MA, Grodzinsky AJ. Mechanical injury of cartilage explants causes specific time-dependent changes in chondrocyte gene expression. Arthritis Rheum. 2005;52: 2386–2395. doi:10.1002/art.21215

34.	Sui Y, Lee JH, DiMicco MA, Vanderploeg EJ, Blake SM, Hung HH, et al. Mechanical injury potentiates proteoglycan catabolism induced by interleukin-6 with soluble interleukin-6 receptor and tumor necrosis factor α in immature bovine and adult human articular cartilage. Arthritis Rheum. 2009;60: 2985–2996. doi:10.1002/art.24857

35.	Brouillette MJ, Ramakrishnan PS, Wagner VM, Sauter EE, Journot BJ, McKinley TO, et al. Strain-dependent oxidant release in articular cartilage originates from mitochondria. Biomech Model Mechanobiol. 2014;13: 565–572. doi:10.1007/s10237-013-0518-8

36.	Martin JA, McCabe D, Walter M, Buckwalter JA, McKinley TO. N-acetylcysteine inhibits post-impact chondrocyte death in osteochondral explants. J Bone Jt Surg - Ser A. 2009;91: 1890–1897. doi:10.2106/JBJS.H.00545

37.	Goodwin W, McCabe D, Sauter E, Reese E, Walter M, Buckwalter JA, et al. Rotenone prevents impact-induced chondrocyte death. J Orthop Res. 2010;28: 1057–1063. doi:10.1002/jor.21091

38.	Delco ML, Bonnevie ED, Szeto HS, Bonassar LJ, Fortier LA. Mitoprotective therapy preserves chondrocyte viability and prevents cartilage degeneration in an ex vivo model of posttraumatic osteoarthritis. J Orthop Res. 2018;36: 2147–2156. doi:10.1002/jor.23882

39.	Eskelinen ASA, Tanska P, Florea C, Orozco GA, Julkunen P, Grodzinsky AJ, et al. Mechanobiological model for simulation of injured cartilage degradation via proinflammatory cytokines and mechanical. PLoS Comput Biol. 2020;16: 1–25. doi:10.1371/journal.pcbi.1007998

40.	Esrafilian A, Stenroth L, Mononen ME, Tanska P, Avela J, Korhonen RK. EMG-Assisted Muscle Force Driven Finite Element Model of the Knee Joint with Fibril-Reinforced Poroelastic Cartilages and Menisci. Sci Rep. 2020;10: 1–16. doi:10.1038/s41598-020-59602-2

41.	Orozco GA, Eskelinen ASA, Kosonen JP, Tanaka MS, Yang M, Link TM, et al. Shear strain and inflammation-induced fixed charge density loss in the knee joint cartilage following ACL injury and reconstruction: A computational study. J Orthop Res. 2021; 0–18. doi:10.1002/jor.25177

42.	Orozco GA, Bolcos P, Mohammadi A, Tanaka MS, Yang M, Link TM, et al. Prediction of local fixed charge density loss in cartilage following ACL injury and reconstruction: A computational proof-of-concept study with MRI follow-up. J Orthop Res. 2020. doi:10.1002/jor.24797

43.	Swärd P, Frobell R, Englund M, Roos H, Struglics A. Cartilage and bone markers and inflammatory cytokines are increased in synovial fluid in the acute phase of knee injury (hemarthrosis) - a cross-sectional analysis. Osteoarthr Cartil. 2012;20: 1302–1308. doi:10.1016/j.joca.2012.07.021

44.	Eskelinen ASA, Florea C, Tanska P, Hung HHK, Frank EH, Mikkonen S, et al. Cyclic loading regime considered beneficial does not protect injured and interleukin-1-inflamed cartilage from post-traumatic osteoarthritis. J Biomech. 2022;141: 111181. doi:10.1016/j.jbiomech.2022.111181

45.	Schuerwegh AJ, Dombrecht EJ, Stevens WJ, Van Offel JF, Bridts CH, De Clerck LS. Influence of pro-inflammatory (IL-1α, IL-6, TNF-α, IFN-γ) and anti-inflammatory (IL-4) cytokines on chondrocyte function. Osteoarthr Cartil. 2003;11: 681–687. doi:10.1016/S1063-4584(03)00156-0

46.	López-Armada MJ, Caramés B, Martín MA, Cillero-Pastor B, Lires-Dean M, Fuentes-Boquete I, et al. Mitochondrial activity is modulated by TNFα and IL-1β in normal human chondrocyte cells. Osteoarthr Cartil. 2006;14: 1011–1022. doi:10.1016/j.joca.2006.03.008

47.	Struglics A, Larsson S, Kumahashi N, Frobell R, Lohmander LS. Changes in cytokines and aggrecan ARGS neoepitope in synovial fluid and serum and in C-terminal crosslinking telopeptide of type II collagen and N-terminal crosslinking telopeptide of type i collagen in urine over five years after anterior cruciate ligame. Arthritis Rheumatol. 2015;67: 1816–1825. doi:10.1002/art.39146

48.	Riegger J, Brenner RE. Evidence of necroptosis in osteoarthritic disease: investigation of blunt mechanical impact as possible trigger in regulated necrosis. Cell Death Dis. 2019;10. doi:10.1038/s41419-019-1930-5

49.	Riegger J, Huber-Lang M, Brenner RE. Crucial role of the terminal complement complex in chondrocyte death and hypertrophy after cartilage trauma. Osteoarthr Cartil. 2020;28: 685–697. doi:10.1016/j.joca.2020.01.004

50.	Tew SR, Kwan APL, Hann A, Thomson BM, Archer CW. The reactions of articular cartilage to experimental wounding: Role of apoptosis. Arthritis Rheum. 2000;43: 215–225. doi:10.1002/1529-0131(200001)43:1<215::AID-ANR26>3.0.CO;2-X

51.	Merrild NG, Holzmann V, Ariosa-Morejon Y, Faull PA, Coleman J, Barrell WB, et al. Local depletion of proteoglycans mediates cartilage tissue repair in an ex vivo integration model. Acta Biomater. 2022;149: 179–188. doi:10.1016/j.actbio.2022.06.032

52.	Li S, Cao J, Caterson B, Hughes CE. Proteoglycan metabolism, cell death and Kashin-Beck Disease. Glycoconj J. 2012;29: 241–248. doi:10.1007/s10719-012-9421-2

53.	DiMicco MA, Sah RL. Dependence of cartilage matrix composition on biosynthesis, diffusion, and reaction. Transp Porous Media. 2003;50: 57–73. doi:10.1023/A:1020677829069

54.	DiMicco MA, Patwari P, Siparsky PN, Kumar S, Pratta MA, Lark MW, et al. Mechanisms and Kinetics of Glycosaminoglycan Release Following In Vitro Cartilage Injury. Arthritis Rheum. 2004;50: 840–848. doi:10.1002/art.20101

55.	Wang P, Tortorella M, England K, Malfait AM, Thomas G, Arner EC, et al. Proprotein Convertase Furin Interacts with and Cleaves Pro-ADAMTS4 (Aggrecanase-1) in the trans-Golgi Network. J Biol Chem. 2004;279: 15434–15440. doi:10.1074/jbc.M312797200

56.	Cao J, Li S, Shi Z, Yue Y, Sun J, Chen J, et al. Articular cartilage metabolism in patients with Kashin-Beck Disease: an endemic osteoarthropathy in China. Osteoarthr Cartil. 2008;16: 680–688. doi:10.1016/j.joca.2007.09.002

57.	Huang L, Riihioja I, Tanska P, Ojanen S, Palosaari S, Kröger H, et al. Early changes in osteochondral tissues in a rabbit model of post-traumatic osteoarthritis. J Orthop Res. 2021; 1–12. doi:10.1002/jor.25009

58.	Williams A, Oppenheimer RA, Gray ML, Burstein D. Differential recovery of glycosaminoglycan after IL-1-induced degradation of bovine articular cartilage depends on degree of degradation. Arthritis Res Ther. 2003;5: 97–105. doi:10.1186/ar615

59.	Torzilli PA, Bhargava M, Park S, Chen CTC. Mechanical load inhibits IL-1 induced matrix degradation in articular cartilage. Osteoarthr Cartil. 2010;18: 97–105. doi:10.1016/j.joca.2009.07.012

60.	Li Y, Frank EH, Wang Y, Chubinskaya S, Huang HH, Grodzinsky AJ. Moderate dynamic compression inhibits pro-catabolic response of cartilage to mechanical injury, tumor necrosis factor-α and interleukin-6, but accentuates degradation above a strain threshold. Osteoarthr Cartil. 2013;21: 1933–1941. doi:10.1016/j.joca.2013.08.021

61.	Venäläinen MS, Mononen ME, Salo J, Räsänen LP, Jurvelin JS, Töyräs J, et al. Quantitative Evaluation of the Mechanical Risks Caused by Focal Cartilage Defects in the Knee. Sci Rep. 2016;6: 1–12. doi:10.1038/srep37538

62.	Chery DR, Han B, Li Q, Zhou Y, Heo SJ, Kwok B, et al. Early changes in cartilage pericellular matrix micromechanobiology portend the onset of post-traumatic osteoarthritis. Acta Biomater. 2020;111: 267–278. doi:10.1016/j.actbio.2020.05.005

63.	Hodgkinson T, Kelly DC, Curtin CM, O’Brien FJ. Mechanosignalling in cartilage: an emerging target for the treatment of osteoarthritis. Nat Rev Rheumatol. 2022;18: 67–84. doi:10.1038/s41584-021-00724-w

64.	Coleman MC, Goetz JE, Brouillette MJ, Seol D, Willey MC, Petersen EB, et al. Targeting mitochondrial responses to intra-articular fracture to prevent posttraumatic osteoarthritis. Sci Transl Med. 2018;10: 1–32. doi:10.1126/scitranslmed.aan5372
